# Relationship between serum iron, zinc, calcium, and HIF-1a—comparative analysis of 2 regions and 4 ethnic groups in China

**DOI:** 10.3389/fnut.2024.1433640

**Published:** 2024-07-22

**Authors:** Yan Guo, Zhong-Sheng Lu, Xue-Cheng Zhang, Qiang Zhang, Xiao Liu, Jie Chen, Meng-Lan Zhang

**Affiliations:** ^1^Department of Pathology, Qinghai Provincial People’s Hospital, Xining, China; ^2^Medical College of Soochow University, Suzhou, China; ^3^Department of Neurosurgery, Qinghai Provincial People’s Hospital, Xining, China; ^4^Qinghai Provincial People’s Hospital, Xining, China; ^5^Department of Basic Medical Sciences, The 960th Hospital of PLA, Jinan, China

**Keywords:** high-altitude illness, iron, zinc, calcium, HIF-1α, altitude, race, linear regression analysis

## Abstract

**Background:**

Altitude illness has serious effects on individuals who are not adequately acclimatized to high-altitude areas and may even lead to death. However, the individualized mechanisms of onset and preventive measures are not fully elucidated at present, especially the relationship between altitude illness and elements, which requires further in-depth research.

**Methods:**

Fresh serum samples were collected from individuals who underwent health examinations at the two hospitals in Xining and Sanya between November 2021 and December 2021. The blood zinc (Zn), iron (Fe), and calcium (Ca) concentrations, as well as hypoxia-inducible factor 1-alpha (HIF-1α) concentrations, were measured. This study conducted effective sample size estimation, repeated experiments, and used GraphPad Prism 9.0 and IBM SPSS version 19.0 software for comparative analysis of differences in the expression of elements and HIF-1α among different ethnic groups, altitudes, and concentration groups. Linear regression and multiple linear regression were employed to explore the relationships among elements and their correlation with HIF-1α.

**Results:**

This study included a total of 400 participants. The results from the repeated measurements indicated that the consistency of the laboratory test results was satisfactory. In terms of altitude differences, except for Fe (*p* = 0.767), which did not show significant variance between low and high altitude regions, Zn, Ca, and HIF-1α elements all exhibited notable differences between these areas (*p* < 0.0001, *p* = 0.004, and *p* < 0.0001). When grouping by the concentrations of elements and HIF-1α, the results revealed significant variations in the distribution of zinc among different levels of iron and HIF-1α (*p* < 0.05). The outcomes of the linear regression analysis demonstrated that calcium and zinc, iron and HIF-1α, calcium and HIF-1α, and zinc and HIF-1α displayed substantial overall explanatory power across different subgroups (*p* < 0.05). Finally, the results of the multiple linear regression analysis indicated that within the high-altitude population, the Li ethnic group in Sanya, and the Han ethnic group in Sanya, the multiple linear regression model with HIF-1αas the dependent variable and elements as the independent variables exhibited noteworthy overall explanatory power (*p* < 0.05).

**Conclusion:**

The levels of typical elements and HIF-1α in the blood differ among various altitudes and ethnic groups, and these distinctions may be linked to the occurrence and progression of high-altitude illness.

## 1 Background

High altitude illness is caused by prolonged exposure to high altitude/low oxygen environments (1). At altitudes of 2,500 meters or higher, anyone who ascends may develop one of three types of acute high altitude illnesses within 1–5 days (2). These include acute mountain sickness (AMS), which is a non-specific symptom complex including headache, fatigue, dizziness, and nausea (3); high altitude cerebral edema (HACE), a potentially fatal condition characterized by ataxia, altered mental status, and characteristic changes on magnetic resonance imaging (4); and high altitude pulmonary edema (HAPE), a non-cardiogenic pulmonary edema caused by excessive hypoxic pulmonary vasoconstriction, which can be fatal if not promptly recognized and treated (5). These illnesses can develop at any time between a few hours and 5 days after ascent, with severity ranging from mild inconvenience to life-threatening conditions (1). The most important factors in the treatment of acute mountain sickness are acclimatization, stopping further ascent and resting, or beginning descent; supplemental oxygen and medication intervention, and, if conditions allow, the use of portable hyperbaric chambers (6). With the increasing popularity of mountain sports and tourism, better education for at-risk populations is crucial (7). Therefore, identifying the risk factors and high-risk populations for high altitude illness and targeted prevention are particularly important.

The pathogenesis of high-altitude illness is characterized by intricate regulation of various physiological systems (8). Prolonged exposure to high-altitude environments leads to a series of adaptive alterations in response to reduced oxygen levels in the atmosphere (9). In terms of adjustments in the respiratory system, an increased respiratory rate and depth help to enhance oxygen uptake to compensate for reduced oxygen availability at high altitudes (10). As for modifications in the cardiovascular system, a heightened heart rate and cardiac output optimize oxygen delivery in the blood, meeting the oxygen needs of tissues and organs. Additionally, prolonged exposure to high altitudes stimulates an increase in red blood cell count, enhancing the capacity for oxygen transport. Enhanced vasodilation also promotes improved blood flow, thereby enhancing the efficiency of oxygen delivery (11). Failure to adapt to the body’s oxygen demands may result in symptoms of high-altitude illness, such as headache, nausea, vomiting, and fatigue. While the systemic physiological responses to acute hypoxia and the adaptive changes have been extensively documented, the molecular mechanisms underpinning these physiological adjustments remain incompletely elucidated (12, 13). Further investigation is warranted to comprehensively unravel factors including hypoxia tolerance, oxidative stress, inflammatory responses, trace elements, and other pertinent aspects.

Hypoxia-inducible factor 1-alpha (HIF-1α), as an important transcription factor, regulates the expression of multiple genes under low-oxygen conditions, promoting cellular adaptation to low-oxygen environments, and serving as a significant marker for high-altitude illness (14). Currently, it is believed that the regulation of HIF-1α may be linked to the onset and progression of high-altitude illness, but the specific mechanisms and impacts require further research for comprehensive understanding (15, 16). Additionally, abundant research indicates the potential pivotal role of elements in the occurrence and development of high-altitude illness (17, 18). Prolonged residence in high-altitude areas may elevate the body’s demand for certain elements due to their critical involvement in regulating internal physiological processes. Furthermore, iron is a component of hemoglobin, aiding in the transportation of oxygen to various parts of the body, maintaining the body’s energy levels, and participating in cellular respiration and energy metabolism processes. Zinc is crucial for the normal functioning of the immune system, promoting wound healing, DNA synthesis, and cell division. Calcium is a key element in maintaining the health of bones and teeth, participating in physiological processes such as nerve conduction, muscle contraction, and blood clotting. These three elements interact synergistically in the human body to maintain normal function and health (19–21). However, the correlation between elements and hypoxia tolerance has not been reported and requires further in-depth exploration.

Xining, the capital of Qinghai Province, is located on the northeastern edge of the Tibetan Plateau, at an altitude of approximately 2,275 meters (7,464 feet), with a cold semi-arid climate. Sanya, a coastal city at the southern tip of Hainan Island in China, was chosen for a low-altitude comparison with Xining, known for its warm and humid tropical monsoon climate year-round. Contrasting climates and altitudes between Sanya and Xining provide insights into how environmental factors influence the levels of elements and HIF-1α concentrations in different populations. Additionally, the Han Chinese, accounting for about 92% of China’s population, live across various altitudes. The Li ethnic group, one of the oldest indigenous groups on Hainan Island, resides in mountainous areas with unique customs and traditions. The Tibetan people, living in the high-altitude Tibetan Plateau, possess unique physiological traits to cope with low oxygen levels. These three ethnic groups offer insights into genetic and environmental adaptations to high-altitude living, particularly in terms of elements and HIF-1α regulation. In this study, 383 healthy young men of Tibetan, Han, and Li ethnicities from Xining and Sanya were included to investigate the levels and relationships of the hypoxia tolerance marker HIF-1α, and three elements, including zinc (Zn), iron (Fe), and calcium (Ca), in different altitudes and ethnic groups, aiming to provide reference value for the prevention, treatment, and pathogenesis exploration of high-altitude illness.

## 2 Materials and methods

### 2.1 Patients and sample

This study was approved by the ethics committee of Hainan Hospital of the General Hospital of the People’s Liberation Army and Qinghai Provincial People’s Hospital (2021-41) and was conducted in compliance with the Declaration of Helsinki (22). Fresh serum samples were collected from individuals who underwent health examinations at the two hospitals between November 2021 and December 2021, and the blood biochemical tests were conducted in the aforementioned hospitals. The information on Xining and Sanya, as well as details on different ethnic groups, are labeled in Supplementary Figure S1.

The inclusion criteria were as follows:

1. Male, 18–45 years old.
2. Li, Tibetan, or Han ethnicity.
3. Have resided in Sanya or Xining for more than 3 years.
4. Have not left their place of residence in the six months prior to blood sample collection.
5. Self-reported absence of physical discomfort.

The exclusion criteria were:

1. Remaining serum sample after clinical test is less than 1.5 mL.
2. Clinically diagnosed patients with malignant tumors, cor pulmonale, hepatitis, nephritis, immune diseases, severe infections, and other illnesses.
3. All participants included in the study have good physical condition, balanced diet, good digestion, normal bowel movements, healthy lifestyle, and have not suffered from chronic or debilitating diseases recently, nor have they taken any medication.

### 2.2 Sample size calculation

The study estimated the minimum sample size required based on the sample size estimation method described in the literature, as well as the data on serum Zn, Fe, Ca, and HIF-1 reported in previous study (23). The significance level (*α*) was set at 0.05, and the power (*β*) at 0.80. By using the values of *μα* = 1.96 (bilateral) and *μβ* = 1.28 (one side), and incorporating the standard deviation (*σ*) and the effect size (*δ*) into the formula (*n* = 2(*μα* + *μβ*)^2^ *σ*^2^/*δ*^2^), the minimum sample size was calculated. Subsequently, the minimum sample size for this study was determined *a priori* based on preliminary data and expected effect sizes, ensuring adequate power to detect significant differences. Using GPower^1^ to conduct post-hoc power analysis was based on the actual collected data to ensure that the sample size was adequate for detecting significant differences. The power for between-group comparisons was calculated and reported in section.

### 2.3 Elimination of data deviation

The blood zinc (bc2815), blood iron (bc1735), and blood calcium (bc0725) concentration detection kits were procured from Beijing Solebao Technology Co., Ltd. (China), while the human HIF-1α ELISA Kit (ft-p36684r) was obtained from Shanghai FanTai Biotechnology Co., Ltd. (China). To mitigate potential discrepancies arising from differences in instruments, equipment, testing personnel, and testing environment between the two laboratories, a pre-formal test phase involved collecting 10 remaining serum samples from clinical tests conducted at two hospitals. Each sample was then divided into two portions, with one stored in cold storage and the other transported to a different laboratory under cold storage conditions. Subsequently, both laboratories conducted simultaneous testing on the same day upon receiving the samples. The resulting test data was compared and analyzed to assess the consistency of the laboratories’ test results. In cases where significant differences were observed, mean calculations were utilized to ensure result consistency prior to further analysis.

### 2.4 Statistical analysis

All statistical analyses were performed using GraphPad Prism 9.0 and IBM SPSS version 19.0 software. Prior to conducting statistical analysis, normality tests were conducted, and when the data met the normal distribution criteria, repeated measurement paired sample *t*-tests was employed to assess the consistency of blood routine test results from the two laboratories. One-way ANOVA was utilized to evaluate differences in serum Zn, Fe, Ca, and HIF-1α content, in cases where the data did not conform to normal distribution, non-parametric Mann–Whitney *U* tests were applied for analysis. A significance level of *p* < 0.05 was used to determine statistical significance. Linear regression analysis was used to explore the relationships between Zn, Fe, Ca, and HIF-1α. In a regression model, if the tolerance is less than 0.01, the variance inflation factor (VIF) value is greater than 10, and the condition index (CI) value is greater than 30, it indicates that there is multicollinearity among the independent variables.

## 3 Results

### 3.1 Sample size estimation

Based on the calculation results from Table 1 and in consideration of the actual circumstances, it is proposed to include the four groups of Sanya Li ethnic group (SL), Sanya Han ethnic group (SH), Xining Han ethnic group (XH), and Xining Tibetan ethnic group (XZ) in this study, with a total of 400 cases. A total of 400 subjects were enrolled in the study. Among them, 17 patients were excluded from the clinical diagnosis of hepatitis, nephritis, thalassemia, leukemia, severe infection and other diseases. A total of 383 patients were actually included in the study, including 97 cases in the Sanya Li ethnic group, 93 cases in the Sanya Han ethnic group, 95 cases in the Xining Han ethnic group, and 98 cases in the Xining Tibetan ethnic group. The result of post-hoc power analysis indicated that our study had a power of 0.85 for detecting between-group differences, meeting statistical requirements.

**Table 1:** Sample size estimation table for this study.

Index		Ethnic 1		Ethnic 2		*δ*	*σ*	*n*
		$\bar{x}$	*s*1	$\bar{x}$	*s*2			
Zn	(μg mL^−1^)	0.943	0.031	0.783	0.195	0.16	0.14	16
Fe	(μg mL^−1^)	1.637	1.612	1.739	0.623	0.102	1.222	3014.3
Ca	(μg mL^−1^)	85.299	1.119	92.578	2.536	7.279	1.96	1.5
HIF-1α	(ng L^−1^)	343.35	45.32	326.51	23.4	16.84	36.066	96.3

*x*, mean value; *s*, standard deviation; allowable error *δ* = |*x*1 − *x*2|, overall standard deviation *σ* = [(*s*1^2^ + *s*2^2^)/2]^1/2^. HIF-1α, hypoxia-inducible factor 1-alpha.

### 3.2 Repeated test

To avoid measurement errors caused by different trial centers, paired sample *t*-tests were used to test the measurement data of 20 patients. The results indicated that the two laboratories tested 20 samples for Zn (*t* = 1.558, *p* = 0.136), Fe (*t* = 1.613, *p* = 0.123), Ca (*t* = 0.458, *p* = 0.652), and HIF-1 α (*t* = 1.197, *p* = 0.246). These findings suggest that the blood routine test results from the two laboratories are consistent, allowing for further data analysis. The consistency of test results from two laboratories was shown in Supplementary Table S1.

### 3.3 Altitude and radial difference analysis

**Figure 1:**
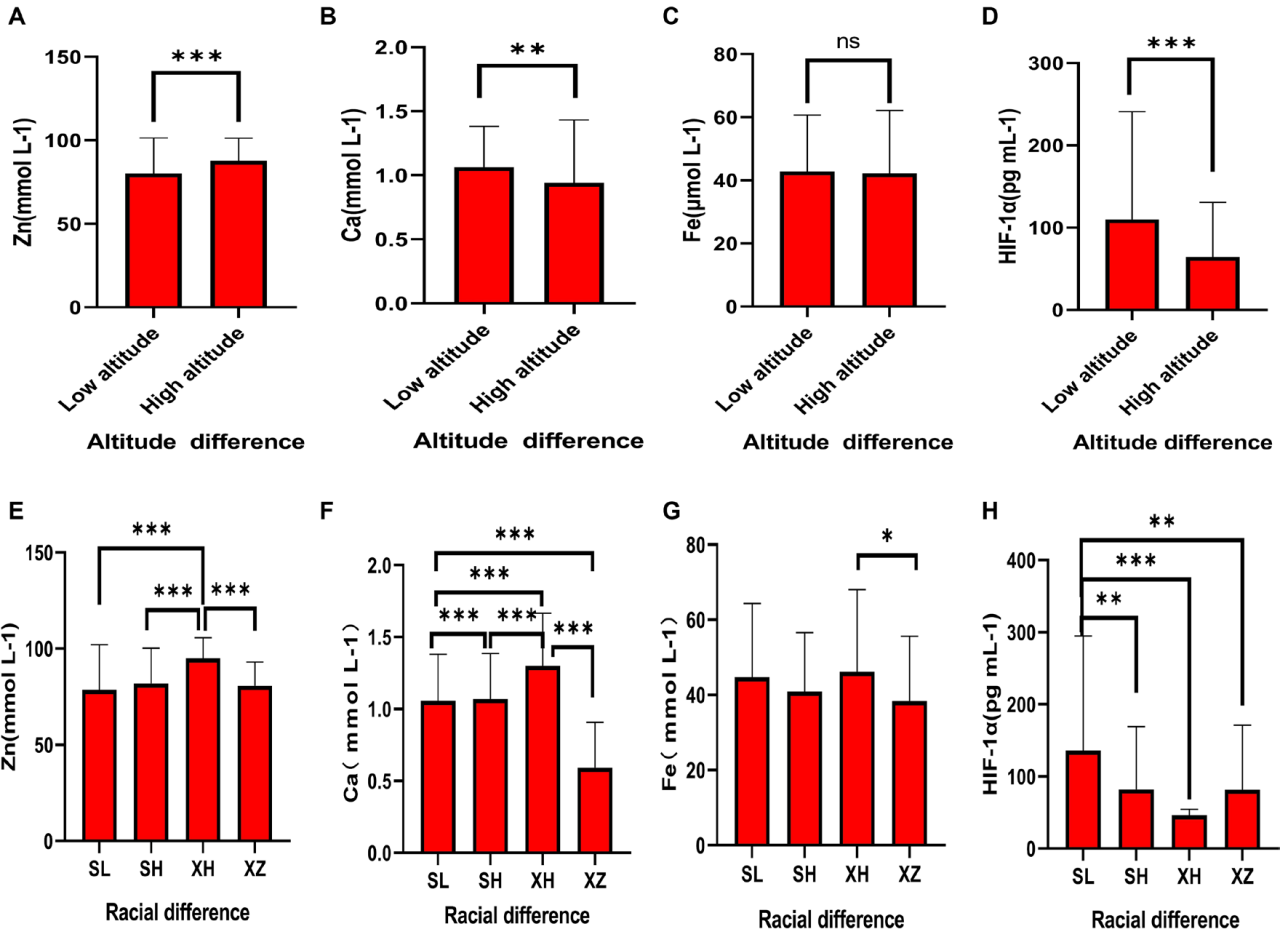
Expression levels of common elements (Zn, Fe, Ca) and HIF-1α in different altitudes and ethnicities. **(A)** Differential expression of Zn in different altitude regions. **(B)** Differential expression of Ca in different altitude regions. **(C)** Differential expression of Fe in different altitude regions. **(D)** Differential expression of HIF-1α in different altitude regions. **(E)** Differential expression of Zn in different ethnicities. **(F)** Differential expression of Ca in different ethnicities. **(G)** Differential expression of Fe in different ethnicities. **(H)** Differential expression of HIF-1α in different ethnicities. “*” indicates a *p*-value less than 0.05, “**” indicates a *p*-value less than 0.01, “***” indicates a *p*-value less than 0.001, and “ns” indicates no statistical significance.

As shown in Figures 1A–D and Table 2, in terms of altitude difference, apart from Fe showing no significant difference between low and high altitude areas, Zn, Ca, and HIF-1α elements all exhibit significant differences between low and high altitude areas. Specifically, Zn content is higher in high-altitude areas, while Ca and HIF-1α content is higher in low-altitude areas. As shown in Figures 1E–H and Table 3, in terms of ethnic differences, there are significant differences in Zn and Ca concentrations among the four ethnic groups, with the highest levels of Zn and Ca found in the high-altitude Xining Han ethnic group and the lowest levels found in the high-altitude Xining Tibetan ethnic group. The Fe concentration in the Xining Han ethnic group is higher than that in the Xining Tibetan ethnic group, and the difference is statistically significant. The HIF-1α concentration in the Sanya Li ethnic group is significantly higher than in the other three groups, with the Xining Han ethnic group having the lowest concentration. The consistency of test results from two laboratories was shown in Supplementary Table S1. The map of the locations of Xining and Sanya, as well as information on different ethnic groups was shown in Supplementary Figure S1.

**Table 2:** Expression levels of common elements (Zn, Fe, Ca) and HIF-1α in different altitudes and ethnicities.

	Low altitude		High altitude		*F*-value	*p*-value
	SL (*n* = 97)	SH (*n* = 95)	XH (*n* = 97)	SH (*n* = 98)		
Zn	78.61 ± 23.51	81.94 ± 18.33	94.95 ± 10.78	80.65 ± 12.36	18.09	<0.0001
Fe	1.058 ± 0.32	1.070 ± 0.32	1.301 ± 0.37	0.593 ± 0.32	78.12	<0.0001
Ca	44.68 ± 1.99	40.88 ± 1.63	46.19 ± 2.24	38.43 ± 1.74	3.433	0.017
HIF-1α	136.20 ± 16.11	82.30 ± 9.011	46.25 ± 0.85	81.90 ± 9.02	12.89	<0.0001

SL, Sanya Li ethnic group; SH, Sanya Han ethnic group; XH, Xining Han ethnic group; XZ, Xining Tibetan ethnic group; HIF-1α, hypoxia-inducible factor 1-alpha.

**Table 3:** Expression levels of common elements (Zn, Fe, Ca) and HIF-1α in different altitudes.

	Low altitude	High altitude	*T*-value	*p*-value
	*n* = 190	*n* = 193		
Zn	80.24 ± 21.14	87.69 ± 13.62	4.108	<0.0001
Ca	1.06 ± 0.32	0.94 ± 0.49	2.983	0.004
Fe	42.82 ± 17.88	42.25 ± 19.95	0.296	0.767
HIF-1α	109.8 ± 9.51	64.35 ± 4.64	4.293	<0.0001

HIF-1α, hypoxia-inducible factor 1-alpha.

### 3.4 Trace element difference analysis

**Figure 2:**
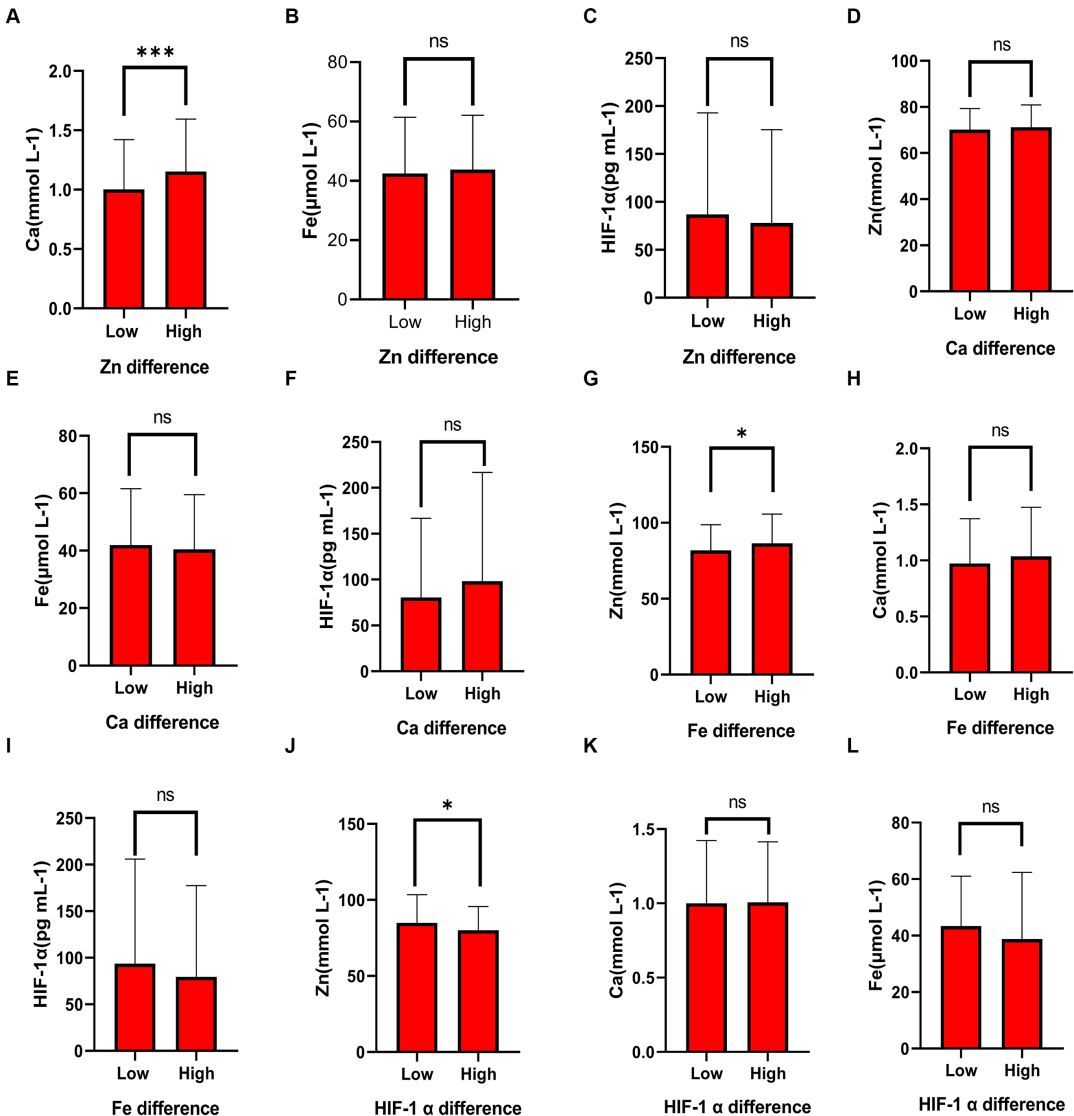
The differential expression of common elements (Zn, Fe, Ca) and HIF-1α in different concentration groups. **(A)** The expression of Ca in the high Zn concentration group and the low Zn concentration group. **(B)** The expression of Fe in the high Zn concentration group and the low Zn concentration group. **(C)** The expression of HIF-1α in the high Zn concentration group and the low Zn concentration group. **(D)** The expression of Zn in the high Ca concentration group and the low Ca concentration group. **(E)** The expression of Fe in the high Ca concentration group and the low Ca concentration group. **(F)** The expression of HIF-1α in the high Ca concentration group and the low Ca concentration group. **(G)** The expression of Zn in the high Fe concentration group and the low Fe concentration group. **(H)** The expression of Ca in the high Fe concentration group and the low Fe concentration group. **(I)** The expression of HIF-1α in the high Fe concentration group and the low Fe concentration group. **(J)** The expression of Zn in the high HIF-1α concentration group and the low HIF-1α concentration group. **(K)** The expression of Ca in the high HIF-1α concentration group and the low HIF-1α concentration group. **(L)** The expression of Fe in the high HIF-1α concentration group and the low HIF-1α concentration group. “*” indicates a *p*-value less than 0.05, “**” indicates a *p*-value less than 0.01, “***” indicates a *p*-value less than 0.001, and “ns” indicates no statistical significance.

To further explore the relationship between elements and HIF-1α, when all populations were grouped by the average concentration of zinc, it was found that calcium concentration significantly increased in the high zinc concentration group, while the differences in iron and HIF-1α concentrations between the high and low zinc concentration groups were not statistically significant (Figures 2A–C and Table 4). Similarly, when all populations were grouped by the average concentration of calcium, the differences in zinc, iron, and HIF-1α concentrations between the two groups were not statistically significant (Figures 2D–F and Table 5). After grouping all populations by the average concentration of iron, it was observed that zinc concentration significantly increased in the high iron concentration group, while the differences in calcium and HIF-1α concentrations between the two groups were not statistically significant (Figures 2G–I and Table 6). Finally, when all populations were grouped by the average concentration of HIF-1α, it was noted that zinc concentration was significantly higher in the low HIF-1α concentration group than in the high HIF-1α concentration group, while the differences in calcium and iron concentrations between the two groups were not statistically significant (Figures 2J–L and Table 7).

**Table 4:** Expression levels of common elements (Fe, Ca) and HIF-1α in the high Zn concentration group and the low Zn concentration group.

	Zn		*T*-value	*p*-value
	Low (*n* = 207)	High (*n* = 176)		
Fe	42.30 ± 18.93	42.81 ± 18.97	0.7937	0.262
Ca	0.71 ± 0.24	1.34 ± 0.31	22.19	<0.0001
HIF-1α	91.35 ± 104.8	81.67 ± 107.4	0.891	0.373

HIF-1α, hypoxia-inducible factor 1-alpha.

**Table 5:** Expression levels of common elements (Zn, Fe) and HIF-1α in the high Ca concentration group and the low Ca concentration group.

	Ca		*T*-value	*p*-value
	Low (*n* = 200)	High (*n* = 183)		
Fe	42.57 ± 19.10	42.49 ± 18.79	0.039	0.969
Zn	77.20 ± 14.38	91.42 ± 18.88	8.333	*p* < 0.0001
HIF-1α	89.62 ± 104.2	83.94 ± 108.0	0.523	0.601

HIF-1α, hypoxia-inducible factor 1-alpha.

**Table 6:** Expression levels of common elements (Zn, Fe) and HIF-1α in the high Fe concentration group and the low Fe concentration group.

	Fe		*T*-value	*p*-value
	Low (*n* = 202)	High (*n* = 181)		
Zn	81.79 ± 16.86	84.46 ± 19.17	2.537	0.012
Ca	0.97 ± 0.40	1.04 ± 0.44	1.467	0.143
HIF-1α	93.64 ± 112.4	79.39 ± 98.04	1.315	0.189

HIF-1α, hypoxia-inducible factor 1-alpha.

**Table 7:** Expression levels of common elements (Zn, Fe, Ca) in the high HIF-1α concentration group and the low HIF-1α concentration group.

	HIF-1α		*T*-value	*p*-value
	Low (*n* = 313)	High (*n* = 70)		
Fe	43.37 ± 17.66	38.80 ± 23.56	1.831	0.068
Zn	84.88 ± 18.54	80.03 ± 15.61	2.034	0.043
Ca	1.00 ± 0.42	1.01 ± 0.41	0.104	0.917

HIF-1α, hypoxia-inducible factor 1-alpha.

### 3.5 Linear regression analysis

**Figure 3:**
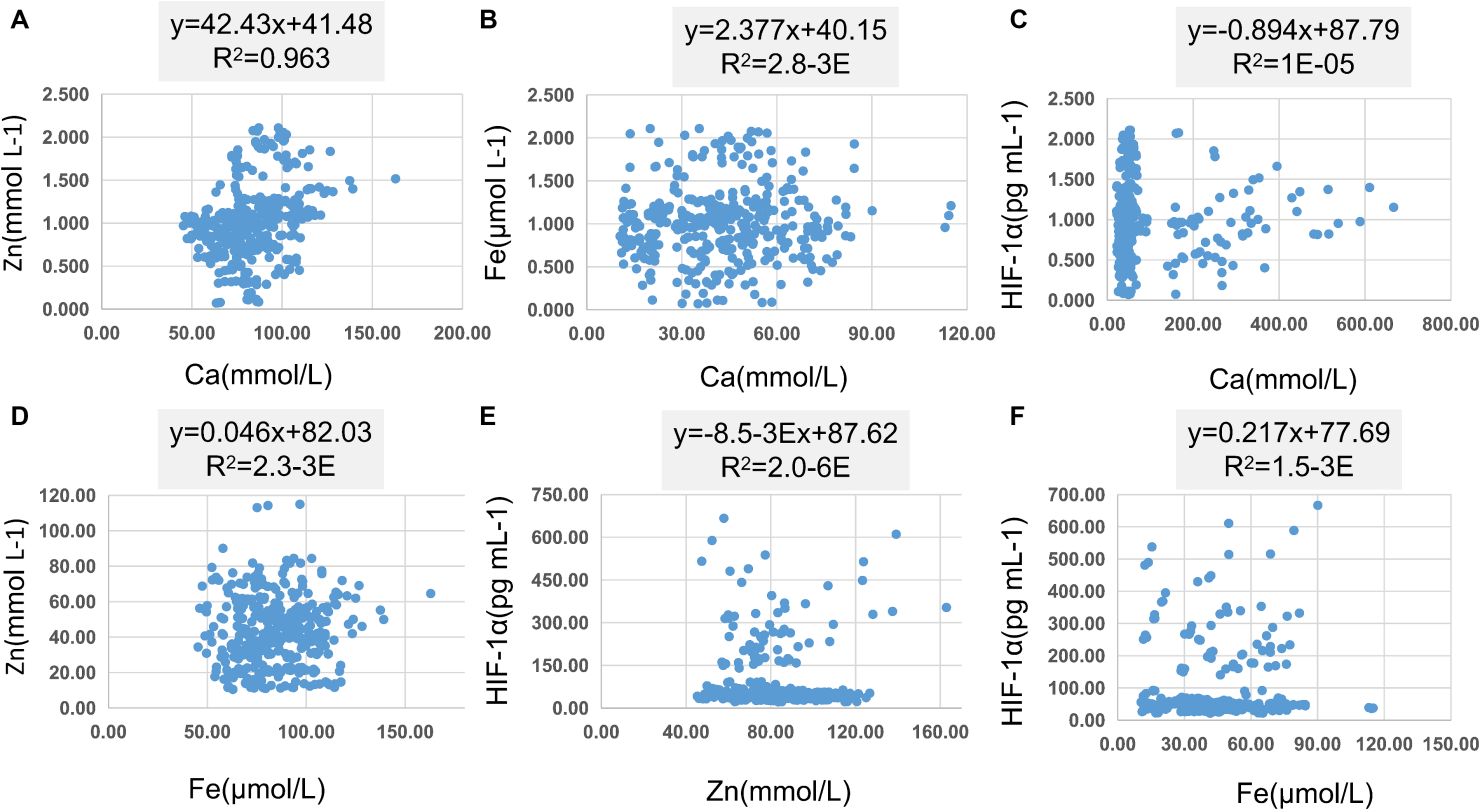
Scatter plot of linear regression between common elements (Zn, Fe, Ca) and HIF-1α. **(A)** Scatter plot of linear regression between Zn and Ca. **(B)** Scatter plot of linear regression between Fe and Ca. **(C)** Scatter plot of linear regression between HIF-1α and Ca. **(D)** Scatter plot of linear regression between Zn and Fe. **(E)** Scatter plot of linear regression between HIF-1α and Zn. **(F)** Scatter plot of linear regression between HIF-1α and Fe.

**Figure 4:**
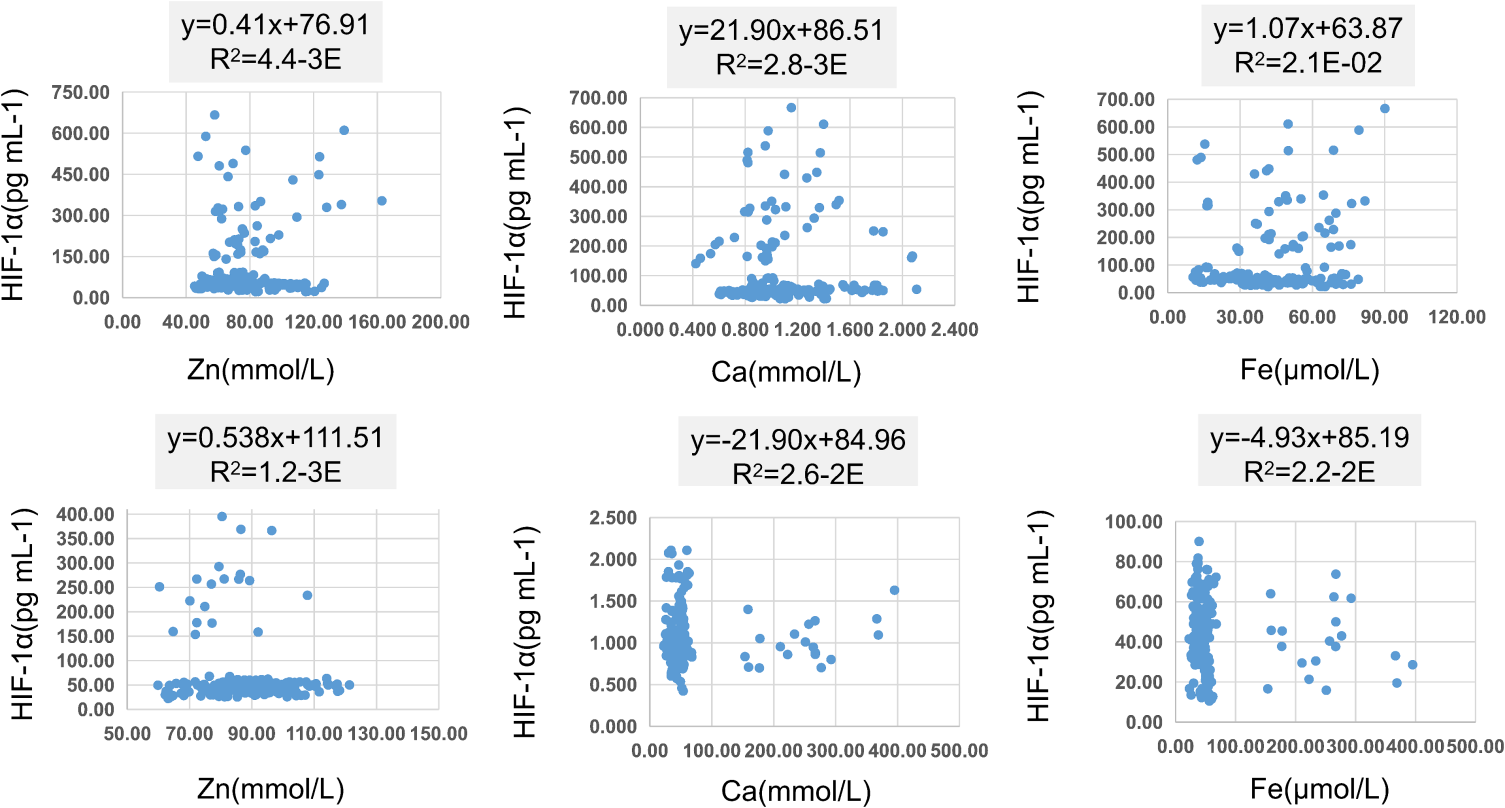
Scatter plot of linear regression between HIF-1α and common elements (Zn, Fe, Ca) in different altitudes. **(A)** Scatter plot of linear regression between HIF-1α and Zn in low-altitude areas. **(B)** Scatter plot of linear regression between HIF-1α and Ca in low-altitude areas. **(C)** Scatter plot of linear regression between HIF-1α and Fe in low-altitude areas. **(D)** Scatter plot of linear regression between HIF-1α and Zn in high-altitude areas. **(E)** Scatter plot of linear regression between HIF-1α and Ca in high-altitude areas. **(F)** Scatter plot of linear regression between HIF-1α and Fe in high-altitude areas.

**Figure 5:**
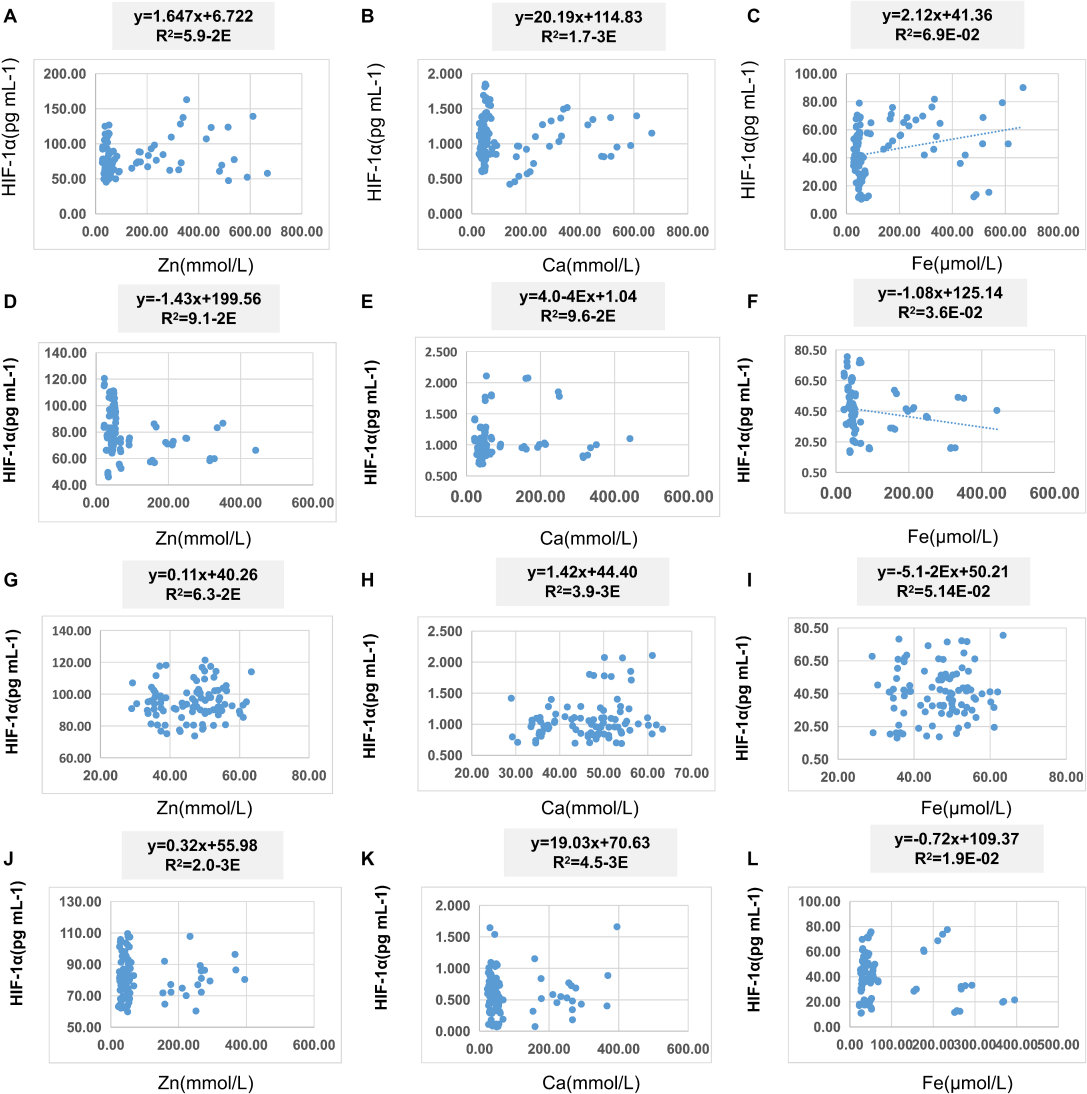
Scatter plot of linear regression between HIF-1α and common elements (Zn, Fe, Ca) in different ethnic groups. **(A)** Scatter plot of linear regression between HIF-1α and Zn in Sanya Li ethnic group. **(B)** Scatter plot of linear regression between HIF-1α and Ca in Sanya Li ethnic group. **(C)** Scatter plot of linear regression between HIF-1α and Fe in Sanya Li ethnic group. **(D)** Scatter plot of linear regression between HIF-1α and Zn in Sanya Han ethnic group. **(E)** Scatter plot of linear regression between HIF-1α and Ca in Sanya Han ethnic group. **(F)** Scatter plot of linear regression between HIF-1α and Fe in Sanya Han ethnic group. **(G)** Scatter plot of linear regression between HIF-1α and Zn in Xining Han ethnic group. **(H)** Scatter plot of linear regression between HIF-1α and Ca in Xining Han ethnic group. **(I)** Scatter plot of linear regression between HIF-1α and Fe in Xining Han ethnic group. **(J)** Scatter plot of linear regression between HIF-1α and Zn in Xining Tibetan ethnic group. **(K)** Scatter plot of linear regression between HIF-1α and Ca in Xining Tibetan ethnic group. **(L)** Scatter plot of linear regression between HIF-1α and Fe in Xining Tibetan ethnic group.

In Table 8, pairwise linear regression analyses were conducted for Zn, Fe, Ca, and HIF-1α content at the overall level. The results indicated a linear correlation between Ca and Zn, with a determination coefficient *R*^2^ of 0.96. However, these relationships were not statistically significant (Figure 3). For low-altitude areas, linear regression analysis was performed for Zn, Ca, Fe, and HIF-1α. The analysis revealed that only the overall explanatory power of the linear regression model between Fe and HIF-1α reached statistical significance (*R*^2^ = 0.02, *p* = 0.04) (Figure 4). In high-altitude areas, the linear regression models for Ca and HIF-1α, as well as Fe and HIF-1α, both demonstrated a significant overall explanatory power (*R*^2^ = 0.03, *p* = 0.02 and *R*^2^ = 0.02, *p* = 0.04, respectively) (Figure 4). When considering regional and ethnic groupings, in the Sanya Li ethnic group, the linear regression models for Zn and HIF-1α, as well as Fe and HIF-1α, both exhibited significant overall explanatory power, with regression equations of (*R*^2^ = 0.06, p = 0.02) and (*R*^2^ = 0.07, *p* = 0.01), respectively. In the Sanya Han ethnic group, only the linear regression model for Zn and HIF-1α showed significant overall explanatory power, with a regression equation of (*R*^2^ = 0.09, *p* = 0.00). For the Xining Han ethnic group, only the linear regression model for Fe and HIF-1α demonstrated significant overall explanatory power, with a regression equation of (*R*^2^ = 0.05, *p* = 0.03). No regression model showed significant overall explanatory power in the Xining Tibetan ethnic group (Figure 5).

**Table 8:** Linear regression between HIF-1α and common elements (Zn, Fe, Ca).

Variable		Person analysis	Model	ANOVA			
X	Y	*R*	*R*2	Regression	Residual	*F* value	*p* value
**Overall**							
Ca	Zn	0.98	0.96	120803.632	4596.40	10013.52	0.00
Ca	Fe	0.05	0.00	379.13	136450.31	1.06	0.30
Ca	HIF-1a	0.00	0.00	53.57	4288563.61	0.00	0.95
Fe	Zn	0.05	0.00	292.47	125107.56	0.89	0.35
HIF-1a	Zn	0.00	0.00	9.09	4288608.10	0.00	0.98
HIF-1a	Fe	0.04	0.00	6416.08	4282201.11	0.57	0.45
**Low altitude areas**							
Zn	HIF-1a	0.07	0.00	14203.96	3235376.10	0.83	0.36
Ca	HIF-1a	0.05	0.00	9241.72	3240338.34	0.54	0.46
Fe	HIF-1a	0.15	0.02	69540.76	3180039.30	4.11	0.04
**High altitude areas**							
Zn	HIF-1a	0.11	0.01	10294.12	830883.20	2.37	0.13
Ca	HIF-1a	0.16	0.03	22254.97	818922.35	5.19	0.02
Fe	HIF-1a	0.15	0.02	18587.39	822589.93	4.32	0.04
**SL group**							
Zn	HIF-1a	0.24	0.06	143947.88	2272238.74	6.02	0.02
Ca	HIF-1a	0.04	0.00	4090.33	2412096.29	0.16	0.69
Fe	HIF-1a	0.26	0.07	167212.23	2248974.39	7.06	0.01
**SH group**							
Zn	HIF-1a	0.30	0.09	63277.64	632194.39	9.11	0.00
Ca	HIF-1a	0.10	0.01	6688.48	688783.54	0.88	0.35
Fe	HIF-1a	0.19	0.04	24832.60	670639.42	3.37	0.07
**XH group**							
Zn	HIF-1a	0.08	0.01	43.45	6374.95	0.63	0.43
Ca	HIF-1a	0.06	0.00	25.23	6393.17	0.37	0.55
Fe	HIF-1a	0.23	0.05	329.56	6088.84	5.03	0.03
**XZ group**							
Zn	HIF-1a	0.04	0.00	1529.28	771904.64	0.19	0.66
Ca	HIF-1a	0.07	0.00	3503.11	769930.80	0.44	0.51
Fe	HIF-1a	0.14	0.02	14645.65	758788.26	1.85	0.18

SL, Sanya Li ethnic group; SH, Sanya Han ethnic group; XH, Xining Han ethnic group; XZ, Xining Tibetan ethnic group; HIF-1α, hypoxia-inducible factor 1-alpha.

### 3.6 Multiple linear regression analysis

As shown in Table 9, using HIF-1α content as the dependent variable and Zn, Fe, and Ca content as the independent variables, multiple linear regression analysis was conducted. The results indicate that there is no issue of multicollinearity in the multiple linear regression models constructed based on data from different altitudes and ethnic groups. However, at the overall level, in low-altitude areas, and in the Xining Han ethnic and Xining Tibetan ethnic groups, the multiple linear regression models did not show a significant overall explanatory power. In high-altitude areas, the multiple linear regression model demonstrated a significant overall explanatory power, with determination coefficients *R*^2^ of 0.04. The coefficients for Zn, Ca, and Fe were −0.19, −17.61, and −0.43, respectively. In the Sanya Li ethnic group, the multiple linear regression model showed a significant overall explanatory power, with determination coefficients *R*^2^ of 0.12. The coefficients for Zn, Ca, and Fe were 1.90, −57.13, and 1.75, respectively. In the Sanya Han ethnic group, the multiple linear regression model demonstrated a significant overall explanatory power, with determination coefficients *R*^2^ of 0.16. The coefficients for Zn, Ca, and Fe were −1.61, 32.04, and −1.21, respectively.

**Table 9:** Multiple linear regression between HIF-1α and common elements (Zn, Fe, Ca).

Variable	Person analysis	Model		ANOVA			Coefficients		Collinearity Statistics		
X	*r*	*R*	*R*2	Regression	*F* value	*p*-value	*B*	Std. Error	Tolerance	VIF	Condition index
**Overall**											
Zn	−0.02	0.05	0.00	9297.41	0.27	0.84	−0.16	0.32	0.89	1.12	4.97
Ca	0.00	0.05	0.00	9297.41	0.27	0.84	0.80	13.74	0.89	1.12	6.90
Fe	0.04	0.05	0.00	9297.41	0.27	0.84	0.23	0.29	0.99	1.01	13.20
**Low altitude areas**											
Zn	0.07	0.16	0.03	87265.51	1.71	0.17	0.26	0.49	0.85	1.18	5.25
Ca	0.05	0.16	0.03	87265.51	1.71	0.17	19.02	32.34	0.84	1.19	9.24
Fe	0.15	0.16	0.03	87265.51	1.71	0.17	1.08	0.53	0.99	1.01	11.52
**High altitude areas**											
Zn	−0.11	0.21	0.04	37626.30	2.95	0.03	−0.19	0.38	0.81	1.23	4.60
Ca	−0.16	0.21	0.04	37626.30	2.95	0.03	−17.61	10.61	0.82	1.23	5.94
Fe	−0.15	0.21	0.04	37626.30	2.95	0.03	−0.43	0.24	0.98	1.02	18.66
**SL group**											
Zn	0.24	0.35	0.12	292325.99	4.27	0.01	1.90	0.84	0.62	1.62	5.08
Ca	0.04	0.35	0.12	292325.99	4.27	0.01	−57.13	60.19	0.63	1.59	9.78
Fe	0.26	0.35	0.12	292325.99	4.27	0.01	1.75	0.80	0.95	1.05	11.05
**SH group**											
Zn	−0.30	0.39	0.16	107806.84	5.44	0.00	−1.61	0.47	0.98	1.02	0.12
Ca	0.10	0.39	0.16	107806.84	5.44	0.00	32.04	26.98	0.98	1.02	0.06
Fe	−0.19	0.39	0.16	107806.84	5.44	0.00	−1.21	0.55	0.98	1.02	0.02
**XH group**											
Zn	0.08	0.24	0.06	359.93	1.80	0.15	0.03	0.08	0.96	1.04	0.16
Ca	0.06	0.24	0.06	359.93	1.80	0.15	1.10	2.33	0.98	1.02	0.05
Fe	−0.23	0.24	0.06	359.93	1.80	0.15	−0.08	0.04	0.98	1.02	0.01
**XZ group**											
Zn	0.04	0.17	0.03	21883.55	0.91	0.44	0.54	0.75	0.96	1.05	0.21
Ca	0.07	0.17	0.03	21883.55	0.91	0.44	1.54	1.75	1.00	1.00	0.09
Fe	−0.14	0.17	0.03	21883.55	0.91	0.44	2.54	2.75	0.96	1.05	0.01

SL, Sanya Li ethnic group; SH, Sanya Han ethnic group; XH, Xining Han ethnic group; XZ, Xining Tibetan ethnic group; HIF-1α, hypoxia-inducible factor 1-alpha.

## 4 Discussion

This study comprehensively analyzed the differences in elements (Zn, Fe, Ca) and HIF-1α expression among different ethnic groups (Tibetan, Li, Han) under different environmental conditions (altitude), revealing the complex interaction of environmental and genetic factors in the regulation of elements in the human body. The significant differences between ethnic groups and altitudes highlight the impact of geographical and sociocultural backgrounds on nutritional status and the potential mechanisms of high-altitude illness. The study found that, apart from Fe showing no significant difference between low and high altitude areas, Zn, Ca, and HIF-1α levels all exhibit significant differences between low and high altitude areas, and there are significant differences in Zn and Ca concentrations among the four ethnic groups. Furthermore, the analysis of trace element differences revealed significant distribution differences in Zn among different concentrations of Fe and HIF-1α. In addition, linear regression suggested that Ca and Zn, Zn and HIF-1α, and Fe and HIF-1α may be linearly correlated in specific populations. Multiple linear regression analysis indicated that in high-altitude areas, specific ethnic groups in Sanya (low altitude) may exhibit multiple linear correlations between elements Zn, Ca, Fe, and HIF-1α. These research findings will provide theoretical support for the role of elements in the occurrence of high-altitude illness.

Fe is an essential constituent of hemoglobin and plays a pivotal role in the genesis and operation of red blood cells, a well-established fact (24). Anemia is widely recognized as a prominent manifestation of iron insufficiency, leading to the synonymous use of iron-deficiency anemia with iron deficiency (21). Furthermore, iron is intricately involved in DNA synthesis, cellular respiration, electron transfer, and overall metabolic processes (25). Recently, the discovery of iron death, a regulated form of cell demise characterized by iron-dependent accumulation of lipid peroxides reaching lethal levels, has been associated with numerous neurodegenerative diseases (26). The significant elevation of human hemoglobin levels with increased altitude of habitation, representing a noteworthy physiological adaptation to enhance oxygen metabolism and acclimate to low-oxygen environments at high altitudes, is well documented (27). However, this study revealed no substantial variance in serum iron concentration between low-altitude and high-altitude cohorts. Notably, among different ethnicities, only the Xining Han ethnic group exhibited significantly higher serum iron levels compared to the Xining Tibetan ethnic group. Investigations have substantiated the substantial iron supply required for augmented red blood cells, while highlighting the potential cytotoxic effects of iron overload, resulting in damage and afflictions in liver, pancreas, and cardiac tissues (27–30). Hepcidin, a hepatic-synthesized small peptide, serves as a critical regulator of iron absorption and internal equilibrium in mammals. Studies have demonstrated a notable decrease in hepcidin in the liver in a murine model of iron deficiency, accompanied by a significant increase in HIF-1α levels (31). Transgenic mice lacking HIF-1α no longer exhibit down-regulation of hepcidin in the liver following iron deficiency, indicating the pivotal role of HIF-1α in governing iron homeostasis and hepcidin regulation (32). This association has been further corroborated in the elegant roundworm, suggesting a potential linear correlation between iron and HIF-1α in specific populations. The adaptive physiological alterations observed in the Han population subsequent to migration to high altitudes due to environmental shifts appear fundamentally distinct from the genetic physiological disparities in minority ethnic groups such as the Li/Tibetan people, who have inhabited diverse altitudes across generations. Consequently, comprehensive consideration of ethnic and geographical factors is imperative in the study of human physiological indicators.

Zn is one of the second most abundant elements in the human body, participating in various important biological processes (33). For example, as a co-factor for 3,000 different proteins, it is involved in the structure and catalytic activity of over 300 enzymes (34). Zinc deficiency can lead to increased oxidative stress, causing damage to DNA, proteins, and lipids (35). Research has also found that young mice deficient in zinc have higher blood-brain barrier permeability after exposure to high levels of oxygen compared to normal zinc levels in young mice (36). Additionally, excessive accumulation of zinc can lead to the loss of tight junction proteins in brain endothelial cells, inducing damage to the blood-brain barrier (37). Zinc plays an important regulatory role in the cell for HIF-1α (38). HIF-1α is an important transcription factor that is activated in cells under low oxygen conditions, promoting the expression of a series of genes to help cells adapt to low oxygen pressure (39). Research indicates that zinc can affect the activity of HIF-1α in multiple ways, including its stability and transcriptional activity. This suggests that the content and homeostasis of zinc in cells are crucial for maintaining the normal function of HIF-1α (38). Therefore, zinc plays an important role in regulating the HIF-1α signaling pathway, which is significant for cellular adaptation to low oxygen environments (40). Our study also found significant differences in the levels of Zn among different altitudes and ethnicities, with higher serum concentrations of Zn observed in high-altitude areas. Additionally, linear regression analysis revealed a linear correlation between Zn and HIF-1α, suggesting that Zn may be closely associated with the occurrence and development of high-altitude illness.

Ca plays an important role in the human body. It is not only a major component of bones and teeth, but also participates in many biological processes, including nerve transmission, muscle contraction, cell signaling, and blood clotting (20). In addition, calcium also plays a crucial role in maintaining the stability and permeability of cell membranes, which is essential for normal cell function (41). Calcium ions (Ca^2+^) can affect the HIF-1α signaling pathway through multiple pathways. Firstly, calcium ions can influence the activity of HIF-1α by regulating its stability. Studies have shown that changes in calcium ion concentration can affect the protein degradation rate of HIF-1α, thereby regulating its level in cells (42). Secondly, calcium ions can also affect the function of HIF-1α by regulating its transcriptional activity. Calcium ions regulate the activity of transcription factors or co-activators associated with HIF-1α, affecting the gene transcription process mediated by HIF-1α (43). These mechanisms make calcium ions play an important role in regulating the ability of cells to adapt to low oxygen environments, thereby affecting cell survival and function (44). Our research has found significant differences in calcium levels among different altitudes and ethnic groups, and there is a significant linear correlation between zinc and calcium. However, further exploration may be needed to understand the involvement of calcium in the pathogenesis of high-altitude illness.

The current study demonstrates several notable strengths. Firstly, a comprehensive analysis was conducted on four ethnic groups residing at two distinct altitudes. The Li nationality, with a population of approximately 1.6 million, has been established on Hainan Island for generations, residing at an average altitude of 3–120 m. Meanwhile, Tibetans, totaling about 4.6 million, have inhabited Qinghai, Tibet, and other plateau regions for centuries, with an average altitude of approximately 4,000 m. Secondly, rigorous measures were implemented to mitigate potential confounding variables. Given the influence of gender, age, and health conditions on serum elements and HIF-1α content, stringent entry criteria were enforced, restricting participation to men who had resided in a specific area for over 3 years, had not traveled to different locations in the 6 months prior to sampling, were aged 18–45 and free from physical discomfort. Additionally, all participants exhibited good physical condition, maintained a balanced diet, normal digestion, regular bowel movements, and a healthy lifestyle, and had not recently suffered from chronic or debilitating illnesses or taken medication, thereby minimizing result deviation. Thirdly, robust statistical analyses were employed, encompassing sample size estimation, repeated experiments, subgroup analysis, linear regression analysis, and thorough validation of results. Nevertheless, our study is subject to certain inherent limitations. Firstly, the study design was based on a cohort study, resulting in a relatively low level of evidence. Secondly, some analytical processes did not undergo further exploration through nonlinear analysis. Lastly, the study solely revealed the correlation between common elements and HIF-1α, without delving into causality and specific mechanisms. It would be beneficial to consider conducting more extensive longitudinal studies to establish causal relationships and explore the underlying mechanisms between common elements and HIF-1α. Additionally, incorporating nonlinear analytical methods could provide further insights into the complex interactions observed in our study.

## 5 Conclusion

In summary, this research identified variations in the concentrations of common elements (e.g., zinc, calcium, and iron) as well as HIF-1α in the serum across diverse altitudes and ethnicities. These variances may be associated with the onset of high-altitude sickness. Through a comprehensive examination of the associations between ethnicity, altitude, and other sociobiological factors on trace element levels, this study provides valuable insights into the nutritional status across different populations. Nevertheless, further investigation is warranted to elucidate the causal correlation and specific mechanisms linking elements to high-altitude illness.

## Data availability statement

The original contributions presented in the study are included in the article/Supplementary material, further inquiries can be directed to the corresponding author.

## Ethics statement

The studies involving humans were approved by the Hainan Hospital of the General Hospital of the People’s Liberation Army and Qinghai Provincial People’s Hospital. The studies were conducted in accordance with the local legislation and institutional requirements. The participants provided their written informed consent to participate in this study.

## Author contributions

YG: Conceptualization, Data curation, Formal analysis, Resources, Writing – original draft, Writing – review & editing. Z-SL: Conceptualization, Formal analysis, Resources, Writing – review & editing. X-CZ: Conceptualization, Formal analysis, Resources, Writing – review & editing. QZ: Conceptualization, Data curation, Project administration, Visualization, Writing – original draft, Writing – review & editing. XL: Data curation, Investigation, Resources, Validation, Writing – original draft. JC: Investigation, Methodology, Project administration, Visualization, Writing – original draft. M-LZ: Data curation, Project administration, Resources, Software, Writing – original draft.

## Footnotes

^1^ http://www.gpower.hhu.de/

## Supplementary material

Supplementary Figure S1 The map of Xining and Sanya, as well as information on different ethnic groups. Xining, the capital of Qing Province, is located on the northeastern edge of the Tibetan Plateau, at an altitude of approximately 2,275 meters, with cold semi-arid climate. Sanya is a coastal city at the southern tip of Hainan Island in China, with an average altitude of 7 meters. It was selected for a low-altitude comparison with Xining, known for its warm and humid tropical monsoon climate throughout the year.
