# Supplementary figures and images for: Relationship between serum iron, zinc, calcium, and HIF-1a—comparative analysis of 2 regions and 4 ethnic groups in China
Source: Front Nutr. 2024 Jul 22;11:1433640. doi: 10.3389/fnut.2024.1433640 (PMC11301158; doi:10.3389/fnut.2024.1433640)

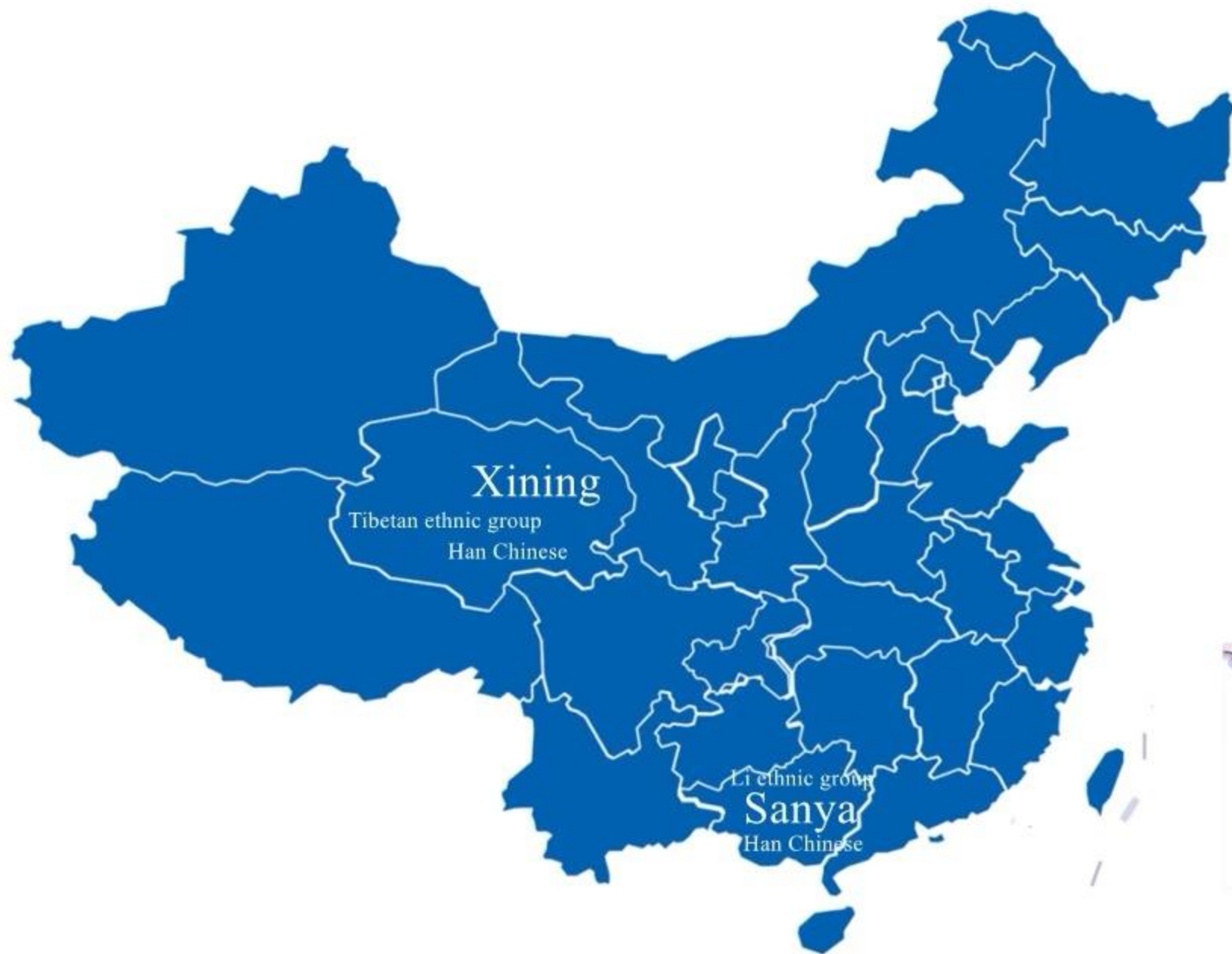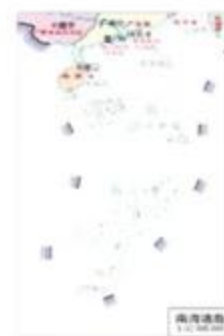

Supplement: Supplementary Figure S1 — The map of Xining and Sanya, as well as information on different ethnic groups. Xining, the capital of Qing Province, is located on the northeastern edge of the Tibetan Plateau, at an altitude of approximately 2,275 meters, with cold semi-arid climate. Sanya is a coastal city at the southern tip of Hainan Island in China, with an average altitude of 7 meters. It was selected for a low-altitude comparison with Xining, known for its warm and humid tropical monsoon climate throughout the year. [file Image_1.pdf]
